# Generation of deletions and precise point mutations in *Dictyostelium discoideum* using the CRISPR nickase

**DOI:** 10.1371/journal.pone.0224128

**Published:** 2019-10-17

**Authors:** Hoshie Iriki, Takefumi Kawata, Tetsuya Muramoto

**Affiliations:** Department of Biology, Faculty of Science, Toho University, Funabashi, Chiba, Japan; University of Sao Paulo, BRAZIL

## Abstract

The CRISPR/Cas9 system enables targeted genome modifications across a range of eukaryotes. Although we have reported that transient introduction of all-in-one vectors that express both Cas9 and sgRNAs can efficiently induce multiple gene knockouts in *Dictyostelium discoideum*, concerns remain about off-target effects and false-positive amplification during mutation detection via PCR. To minimise these effects, we modified the system to permit gene deletions of greater than 1 kb via use of paired sgRNAs and Cas9 nickase. An all-in-one vector expressing the Cas9 nickase and sgRNAs was transiently introduced into *D*. *discoideum*, and the resulting mutants showed long deletions with a relatively high efficiency of 10–30%. By further improving the vector, a new dual sgRNA expression vector was also constructed to allow simultaneous insertion of two sgRNAs via one-step cloning. By applying this system, precise point mutations and genomic deletions were generated in the target locus via simultaneous introduction of the vector and a single-stranded oligonucleotide template without integrating a drug resistance cassette. These systems enable simple and straightforward genome editing that requires high specificity, and they can serve as an alternative to the conventional homologous recombination-based gene disruption method in *D*. *discoideum*.

## Introduction

The social amoebae *Dictyostelium discoideum* has been used as a simple eukaryotic model organism for several decades. This organism is relatively easy to grow as individual cells; however, upon starvation, the cells begin to assemble into chemotaxis-mediated aggregates that ultimately form a multicellular fruiting body that consists of two types of differentiated cells: stalk cells and spore cells. As a consequence, this organism is useful for examining genes involved in fundamental cellular and developmental functions, such as transcriptional regulation, cell migration, phagocytosis and macropinocytosis [1].

Since the genome sequence of *D*. *discoideum* was determined [2], gene knockout mutants have been extensively generated to investigate the functions of genes. Several marker genes, such as those that confer resistance to blasticidin, G418, or hygromycin, have been used to target genes of interest [3]. However, gene knockout methods based on homologous recombination (HR) are sometimes inefficient and time-consuming, especially for generating multiple gene knockouts as the Cre-*loxP* system sequentially knocked out the genes by recycling the drug resistance cassette [4]. Recently, an efficient markerless gene targeting method based on the CRISPR/Cas9 system was developed in *D*. *discoideum* [5, 6]. In this system, the Cas9 nuclease and sgRNAs are simultaneously expressed from an all-in-one vector. The Cas9/sgRNA complex recognises a specific 20-nucleotide target site adjacent to a protospacer adjacent motif (PAM) sequence. The complex induces a double-strand break (DSB) approximately 3 base pairs (bp) upstream of the PAM sequence, and the DNA damage is then repaired via non-homologous end joining (NHEJ) or homology-directed repair (HDR) [7]. NHEJ leaves insertions or deletions (indels) at the cleavage site, and the HDR pathway can introduce sequences encoding a fluorescent protein, tag, or point mutation into the gene of interest [8, 9]. The resulting indels are very likely to disrupt the target gene by introducing frameshift mutations in protein-coding regions.

Despite the great promise and robustness of the CRISPR/Cas9 system, there are two concerns regarding the use of this system in *D*. *discoideum*. First, the targeting specificity is expected to be poor [5, 6]. In mammalian cells that are extensively studied, Cas9 nucleases sometimes cleave off-target sites that share high sequence homology to the target site [10–13]. Refinements to improve targeting specificity to minimise off-target effects are essential. Second, the sensitivity in the screening and validating of the CRISPR-modified indel mutants via mutation-detecting PCR is insufficient in the current system [6]. Unlike the conventional gene knockouts generated via HR-based methods, the indel mutations generated by CRISPR/Cas9 consist of deletions or insertions of one or a few nucleotides in the target region. By designing one of the primers to span the cleavage site, which contains an indel mutation that alters a few nucleotides, it is possible to identify the mutation as PCR amplification is suppressed at the mutated locus. However, the possibility of false-positives due to inefficient PCR amplification cannot be excluded; thus ambiguities remain in the method. Therefore, an accurate mutation detection method in which the lengths of the PCR products generated from the wild-type and mutant templates that are obviously different is required.

To reduce the probability of off-target effects without decreasing the gene targeting efficiency, a Cas9 nickase was developed to introduce target-specific single-strand breaks (nicks) [14–16]. Individual nicks in the genome are repaired with high fidelity [17], while simultaneous production of nicks on both DNA strands by a pair of Cas9 nickases leads to a target-specific DSB that is repaired by NHEJ [18, 19]. The cleavage activity of Cas9 nuclease is mediated by two catalytic domains, RuvC and HNH. These domains were inactivated via point mutations (D10A in RuvC and H840A in HNH) such that the Cas9 nuclease was converted into a Cas9 nickase [15, 20, 21]. The RuvC domain is required for cleavage of the non-target strand; therefore, the D10A mutant only produces a nick in the target strand that contains the PAM sequence. Conversely, the HNH domain normally cleaves the target strand; however, the H840A mutant generates a nick in the strand complementary to the sgRNA. Although designing a pair of sgRNAs for the Cas9 nickase system is more complicated, since two sgRNAs with PAM sequences facing outward must be selected, the double nicking method has been successfully used to reduce off-target effects by 50- to 1,500-fold without reducing the gene targeting efficiency [15, 16, 22]. Using this method, deletions of up to 100 bp were generated in the first report [15, 16], while later reports described deletions of approximately 1 kb [22]. Such long deletions support improved mutation detection due to the larger size differences in the PCR products generated from the WT and mutant lines.

In this study, we developed an efficient and straightforward Cas9 nickase-mediated genome editing system with an all-in-one vector for use in *D*. *discoideum*. The system efficiently generated mutants with deletions longer than 1 kb. Furthermore, by mixing single-stranded oligo DNA (ssODN) with the vector, precise deletions and point mutations were generated without introducing drug resistance cassettes. The newly developed CRISPR nickase system will serve as a powerful and valuable genome modification tool in *D*. *discoideum*.

## Methods

### Cell culture and isolation of transformants

Axenic AX2 cells were cultured at 22°C in HL5 medium or on SM agar plates with *Klebsiella planticola* (recently reclassified as *Raoultella planticola*). Transformation was performed as described previously [6] with minor modifications. Before electroporation, 10 μg of the appropriate CRISPR vector was mixed with cells, and the mixture was transferred to an electroporation cuvette. After the electroporation, the cells were incubated in culture dishes containing 10 ml HL5 medium. To obtain cells transiently expressing Cas9 and the sgRNAs, we replaced the plain HL5 medium with HL5 containing 10 μg/ml of G418 7–24 h after electroporation and then maintained the cells for another 20–42 h. For stable expression of Cas9 and the sgRNAs, the transformed cells were maintained in HL5 containing 10–20 μg/ml of G418 for at least 7 days. The aggregation phenotypes of independent clones were analysed by plating the cells on SM agar plates.

### Construction of the Cas9 nickase-sgRNA expression vector

To construct the tRNA-sgRNA cassette containing two Esp3I (BsmBI) sites, the two BpiI sites in the tRNA-sgRNA cassette in pTM1422 were converted to two Esp3I sites by inserting a pair of annealed oligonucleotides, i.e., 5'-AGCAGGAGACGGGCGTCTCG-3' and 5'-AAACCGAGACGCCCGTCTCC-3'. The resulting vector, pTM1493, was digested with XhoI to insert the XhoI- and SalI-flanked tRNA-sgRNA cassette obtained from pTM1422. After the cloning, the dual tRNA-sgRNA cassettes containing the BpiI and Esp3I sites in pTM1494 were excised via digestion with XhoI and partial digestion with HindIII, and these constructs were then cloned into the XhoI and HindIII sites of the pTM1285 vector [6] to generate a vector (pTM1291) for simultaneously expressing Cas9 nuclease and two sgRNAs. We also generated a Cas9 nuclease and dual sgRNA expression vector with a G418 resistance marker driven by the *coaA* promoter (pTM1372) (S1A Fig).

To construct a vector for constitutively expressing Cas9 and sgRNAs, the extrachromosomal vector pDM1208 [23] was used. The BglII- and SpeI-flanked Cas9-NLS-GFP fragment was obtained by digesting the pTM1285 vector with these enzymes, and the resulting fragment was then ligated into the BglII and SpeI sites of the pDM344 shuttle vector [24]. The resulting Cas9-NLS-GFP expression cassette was subsequently excised via partial digestion at the NgoMIV restriction sites. The NgoMIV-flanked Cas9 cassette and the XhoI/HindIII-flanked tRNA-sgRNA cassette obtained from pTM1493 were cloned into the pDM1208 vector via the NgoMIV or XhoI and HindIII restriction sites, respectively. As a result, the Cas9/sgRNA constitutive expression vector, pTM1351, was generated.

The D10A-substituted Cas9 was created as follows. The D10A fragment of dCas9 was excised from a dCas9-NLS-GFP vector, pTM809, using BglII/KpnI, and the fragment was then inserted into the Cas9 and dual sgRNA expression vector (pTM1291) via the BglII/KpnI sites, yielding the Cas9 nickase and dual sgRNA expression vector, pTM1331 (S1B Fig). To construct the vector constitutively expressing the Cas9 nickase, the BglII/KpnI-flanked D10A fragment was cloned into pTM1351 via the BglII/KpnI sites. The resulting Cas9 nickase vector, pTM1355, contained two Esp3I sites within the tRNA-sgRNA cassette; thus Esp3I was used to insert sgRNAs (S1C Fig).

To construct the dual sgRNA expression vector for one-step cloning, the two Esp3I sites in the tRNA-sgRNA cassette were converted into two BpiI sites via addition of a pair of annealed oligonucleotides, i.e., 5'-AGCAGGTCTTCGGGAAGACG-3' and 5'-AAACCGTCTTCCCGAAGACC-3'. An XhoI linker was then inserted in the HindIII site at the end of the sgRNA. The XhoI flanked tRNA-sgRNA cassette containing the new BpiI sites was ligated into the XhoI site of the tRNA-sgRNA cassette with the original BpiI sites. The resulting vector, pTM1544, was used to simultaneously insert two sgRNAs via one-step cloning (S1D Fig). These plasmids will be made available to all researchers via NBRP Nenkin and other stock centres.

### Designing and cloning of sgRNA sequences

We used Cas-Designer to select 20-nucleotide target sequences in the *pkaC* gene [25]. The sgRNA sequences were synthesised as a pair of forward and reverse oligonucleotides, as shown in Table 1, and these sequences were then annealed to form a double-stranded structure. Each pair of annealed oligonucleotides contained unique 4-nucleotide overhangs compatible with the ends of BpiI or Esp3I digested vectors. The CRISPR vectors contained two BpiI or Esp3I sites between the tRNA and sgRNA scaffold; therefore, the Golden Gate digestion-ligation reaction was used to insert the pair of annealed oligonucleotides as described previously [5, 6]. For one-step cloning with the dual sgRNA expression vector, a pair of oligonucleotides with appropriate overhangs (5 μM each) were heated at 95°C for 5 minutes and then slowly cooled to 25°C using a thermal cycler. The annealed oligonucleotides were phosphorylated by mixing 0.5 μM solutions of each primer with 0.2 μl of T4 PNK (NEB) in 10.0 μl reactions that were then incubated at 37°C for 30 minutes. Pairs of sgRNA duplexes (0.26 μl each) were ligated into the pTM1544 vector (40 ng) via one-step Golden Gate digestion/ligation reactions using 0.4 μl T4 DNA ligase and 0.3 μl BpiI (Thermo) in 8.0 μl reactions. The mixtures were placed in a thermal cycler and subjected to eight cycles of 37°C for 5 minutes and 16°C for 17 minutes. After completion of the cycles, an additional BpiI digestion was performed at 37°C for 60 minutes to prevent contamination of the bacterial transformations with vectors lacking the sgRNA sequences. Correct assembly of dual-sgRNA constructs was confirmed via colony PCR using the following primers: Neo 5'- TCCTGCAGTTCATTCAGGGC-3' and B1-2 primers for the first target, and GFP 5'- TGGAAGCGTTCAACTAGCAG-3' and T1 primers for the second target (Table 1). Correct insertion was also confirmed via Sanger sequencing from the Neo primer.

**Table 1:** List of oligonucleotides used to generate the sgRNA vectors.

Name	Position	Direction	Sequence (5'- to -3')
B1	216	-	agcaTTGCTGAGGTCACAGGACTA
			aaacTAGTCCTGTGACCTCAGCAA
B1-2	216	-	gagcaTTGCTGAGGTCACAGGACTAg
			taaacTAGTCCTGTGACCTCAGCAAt
T1	309	+	agcaATCACCTCTATTTCATATCT
			aaacAGATATGAAATAGAGGTGAT
T2	664	+	agcaCAACAACACCTACATCAAGA
			aaacTCTTGATGTAGGTGTTGTTG
T3	745	+	agcaACAACAAATCCTCATACATC
			aaacGATGTATGAGGATTTGTTGT
B2	883	-	agcaGACTGTTGTTGTTGTCTGAT
			aaacATCAGACAACAACAACAGTC
T4	1026	+	agcaTGGTACTGGAACATTTGGAA
			aaacTTCCAAATGTTCCAGTACCA
T5	1390	+	agcaAATTTGTTGATCGATAATCA
			aaacTGATTATCGATCAACAAATT
T6	1512	+	agcaAAGCAAAGGTCATGGTAAAG
			aaacCTTTACCATGACCTTTGCTT
T7	1531	+	agcaGCGGTCGATTGGTGGGCACT
			aaacAGTGCCCACCAATCGACCGC
T8	1893	+	agcaTTATATCAGAGAAGAGATAA
			aaacTTATCTCTTCTCTGATATAA

The upper and lower case letters indicate the target sequence and overhangs for the Golden Gate cloning, respectively. The name and location of each target sequence are also shown in Fig 2A. The number shown in a given position is a relative number indicating ATG +1. The sgRNA sequences in the sense and antisense orientations are indicated as + and -, respectively.

## Results

### CRISPR/Cas9-mediated deletion generation in *D*. *discoideum*

**Fig 1:**
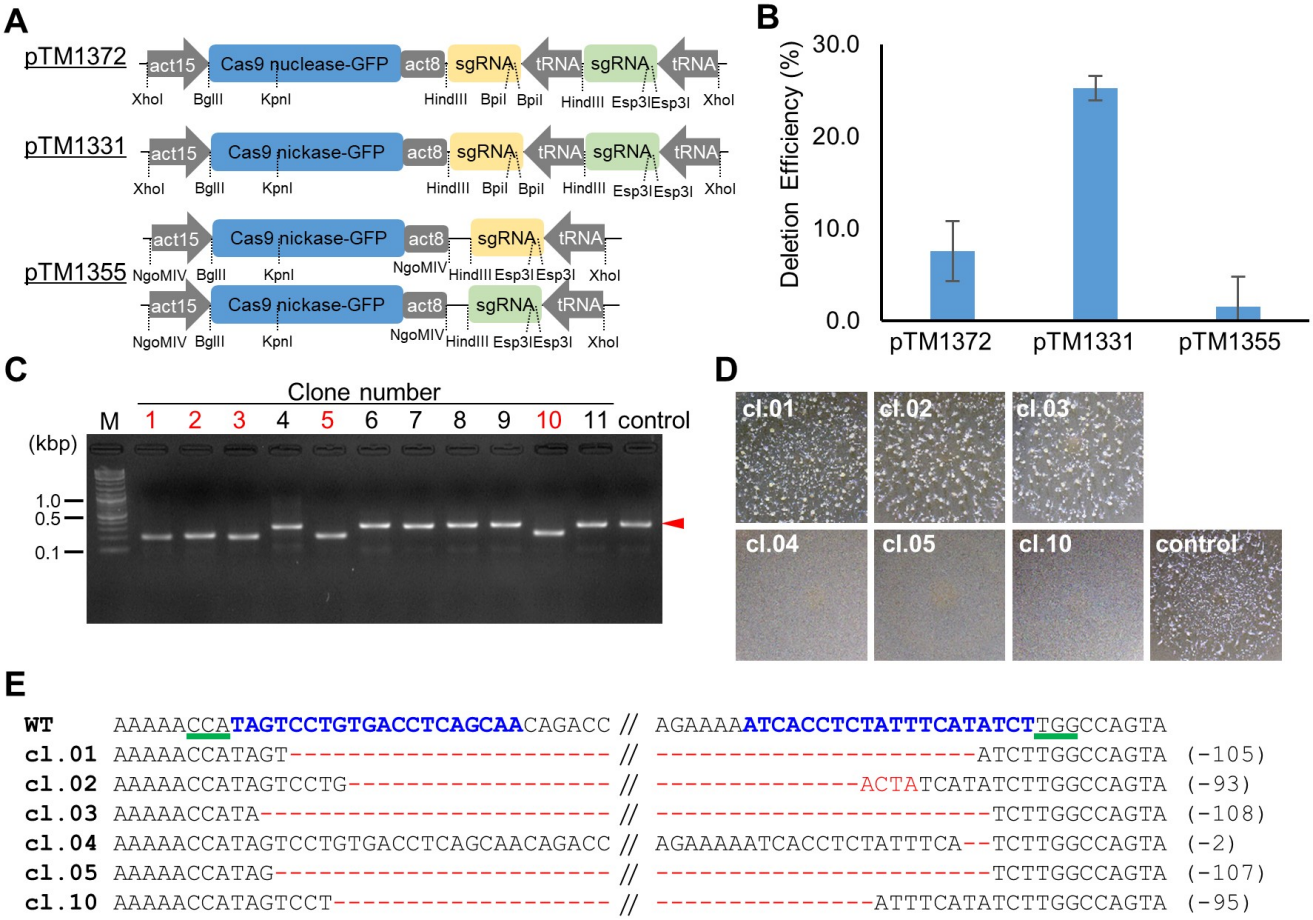
CRISPR/Cas9-mediated generation of deletion mutations in *Dictyostelium*. (A) Schematic overview of the CRISPR vectors designed to generate deletion mutations. sgRNA sequences were synthesised as pairs of oligonucleotides and integrated into the vectors using the Golden Gate assembly method. act15, act15 promoter; act8, act8 terminator; tRNA, isoleucine tRNA. (B) CRISPR-mediated deletion efficiencies. Single clones grown on SM agar plates were randomly selected and tested for deletions via PCR amplification of the target region. The error bars show the standard error of the mean based on three independent biological repeats. (C) Genomic deletions in pTM1331-expressing cells. PCR products generated using primers flanking the target sites are shown. A control reaction using AX2 cells is shown in the rightmost lane. Red arrowhead indicates PCR products found in the control. (D) Aggregation phenotypes of the mutants shown in Fig 1C. Single clones were seeded on a bacterial lawn and imaged after 4 days with a stereoscopic microscope. (E) The sequencing results of the deletion regions. The wild-type (WT) sequence of *pkaC* and six independent mutated sequences are shown. The sequence used as the target is shown in blue and the PAM sequences are shown by green underscores. The mutated nucleotides are in red, and the numbers in parentheses indicate the number of deleted nucleotides.

The CRISPR/Cas9 system is known to induce unintended mutations at off-target sites that share sequence similarities with the target site. By contrast, Cas9 nickase-generated nicks at off-target sites are repaired with high fidelity and are unlikely to recruit the NHEJ machinery. Additionally, Cas9 nuclease-mediated indel mutagenesis is not suitable for creating long deletions since insertions or deletions of only a few nucleotides are often introduced. Thus, we attempted to develop a double nicking Cas9 nickase system to create a genome editing technology for generating long deletions in *D*. *discoideum* cells. To this end, we used Cas9 nickase (D10A) instead of Cas9 nuclease. We modified a previously reported CRISPR/Cas9 system in *D*. *discoideum* [6] and constructed three expression constructs (pTM1372, pTM1331 and pTM1355) (Fig 1A) to compare the deletion efficiency. We selected the T1 and B1 sequences of the *pkaC* gene as the first targets to generate deletions (Table 1). The gene encodes the catalytic subunit of the cAMP-dependent protein kinase (PKA) and the null mutants exhibit an aggregation minus phenotype [26, 27]. As the pTM1372 and pTM1331 vectors possess two tRNA-sgRNA cassettes with BpiI and Esp3I restriction sites, T1 and B1 targeting sequences were sequentially inserted into each restriction site via two-step cloning. By contrast, the pTM1355 vector contains a single tRNA-sgRNA cassette; thus the T1 and B1 sgRNA sequences were cloned separately into the vector. The two obtained expression vectors were simultaneously introduced into cells via electroporation.

To analyse the deletions in the *pkaC* locus generated via transient expression of the sgRNAs and Cas9 nuclease from the pTM1372 vector, we amplified the target region via PCR. We observed that nearly 10% of the clones carried deletions in the locus (Fig 1B). Since aggregation-negative clones without detectable genomic deletions were observed at a higher frequency on SM agar plates, indel mutations were more likely than complete deletions when using the Cas9 nuclease. By contrast, nearly 30% of the clones showed genomic deletions in the target region when the sgRNAs and Cas9 nickase were transiently expressed from pTM1331. Notably, when the two target sequences were expressed using separate pTM1355 vectors, the deletion efficiency was lower than that obtained with the dual sgRNA expression vector, pTM1331, even though the pTM1355 vectors were constitutively maintained extrachromosomally (Fig 1B). Deletions of approximately 100 bp were clearly detected following electroporation in cells that transiently carried the pTM1331 vector (Fig 1C). The phenotypes of these clones were categorised as either aggregation-positive or aggregation-negative. We obtained over 10,000 clones from independent transformations, and ~40% of the clones were identified as aggregation-negative. The three clones, #01, #02, and #03, shown in Fig 1D were aggregation-positive despite the fact that deletions were detected via PCR, as shown in Fig 1C. Therefore, the genomic region of the *pkaC* gene was sequenced to determine whether the deletions occurred within the target region. The results confirmed that all of the clones had the deletions predicted by the PCR (Fig 1E). However, the deletions were in multiples of three nucleotides, resulting in the deletion of several dozen amino acids from the proteins. Since the deleted region is not essential for ATP binding or protein kinase activity, the protein is predicted to retain most of its essential activity. By contrast, clones #05 and #10 showed approximately 100 bp deletions and the aggregation-negative phenotype, indicating that the Cas9 nickase-generated deletions disrupted the gene’s function. Therefore, deletions of approximately 100 bp were successfully generated by using the Cas9 nickase, and the accuracy of the mutation detection PCR was clearly improved due to the improved ability to detect the differences in the band sizes from the wild-type and mutant lines.

### Efficiency of Cas9 nickase-mediated generation of long deletions

**Fig 2:**
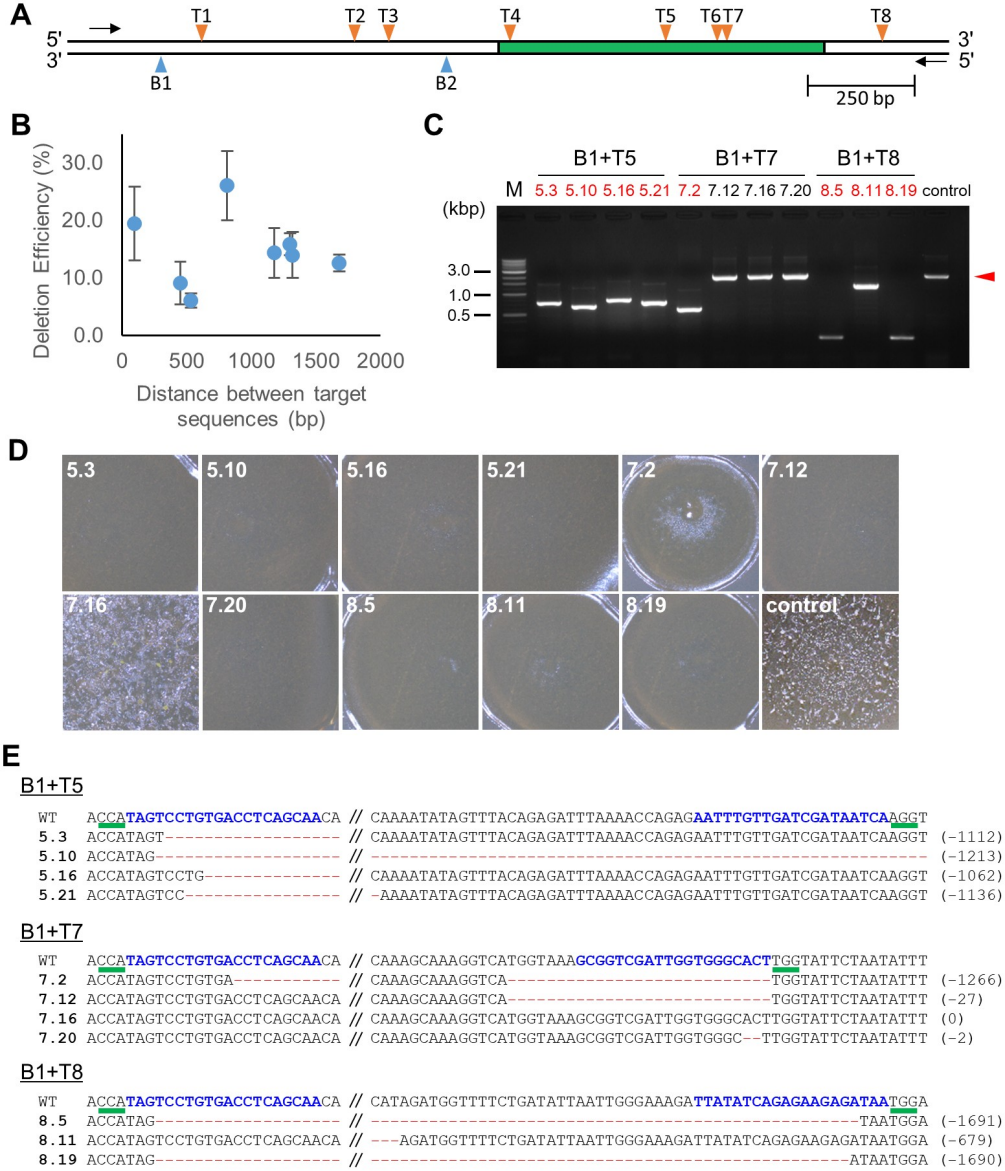
Long genomic deletions generated via use of paired Cas9 nickases. (A) Target sites in the *pkaC* gene locus. The position of each target site is indicated by an arrowhead. The arrows indicate the locations of the PCR primers used to detect the deletions. The box in green represents the protein kinase domain predicted by UniProt. (B) Analysis of the deletion efficiencies of paired Cas9 nickases. A total of 22 independent clones were isolated from individual transformations and scored for the presence of deletions via PCR. The error bars show the standard error of the mean based on three independent transformations. (C) Representative genomic deletions detected by PCR. The arrowhead indicates product size found in the control (the AX2 genome). Clone numbers are shown in red and black, and indicate obvious and unapparent deletions, respectively. (D) Aggregation phenotypes of the mutants. (E) Representative DNA sequences of the target region. The PAM sequences are shown by green underscores. The sgRNA-matching sequences are shown in blue. The mutated nucleotides are in red, and the numbers in parentheses indicate the number of deleted nucleotides.

Although we showed that deletions of approximately 100 bp could be generated with high efficiency, some of the deletion mutants without frameshifts retained the gene function required for cell aggregation. Since longer deletions are more likely to cause loss of function, we examined whether it is possible to generate long deletions of 1000 bp or more. For this assay, we designed nine sgRNAs covering about 1700 bp of the *pkaC* gene (Fig 2A). We selected a pair of sgRNAs with outward facing PAM sequences and then cloned them into the pTM1331 vector via two-round cloning. After transient expression of Cas9 nickase and the sgRNAs, we performed PCR to detect deletions in the target region. We found that all eight pairs of sgRNAs induced genomic deletions of up to 1.7 kb within the gene. No obvious decrease in deletion efficiency dependent on the spacing of a pair of sgRNAs was observed (Fig 2B & 2C). These data showed that Cas9 nickase-mediated generation of long deletions occurred with high efficiency.

Since long deletion mutants were obtained, we examined whether the aggregation defect was observed as frequently in these mutants as in the short deletion mutants. As shown in Fig 2D, all of the deletion mutants showed the aggregation-minus phenotype and had a smooth surface. In clones 7.12 and 7.20, aggregation defects were observed even though no obvious deletion was detected via PCR. The nucleotide sequences of these mutants revealed that 27 bp and 2 bp deletions were generated, respectively (Fig 2E). The 7.16 clone, in which cell aggregation was observed, had the wild-type genomic sequence, indicating that it was not a CRISPR mutant as expected. The nucleotide sequences of the other deletion mutants were also analysed, and the presence of the expected deletions was confirmed. However, by comparing the target sequences with the deleted regions, it was found that the deletions did not necessarily begin at the target sequence. We propose that this tendency is due to the target sequence rather than differences in the distance between a pair of sgRNAs, as the B1+T8-mediated mutants (clones 8.5 and 8.19), which carried the longest deletions, had deletion ends between the two sgRNAs.

We also analysed the difference in deletion efficiency depending on the combination of target sequences. It is previously reported that two sgRNAs with inward facing PAM sequences did not lead to robust deletion mutagenesis with Cas9 nickase (D10A) [21]. This finding was also true in *D*. *discoideum*. No deletion mutants were detected in three independent experiments in which we introduced T2+B2 target sequences.

### Single-step assembly of a Cas9 nickase vector for dual sgRNA expression

**Fig 3:**
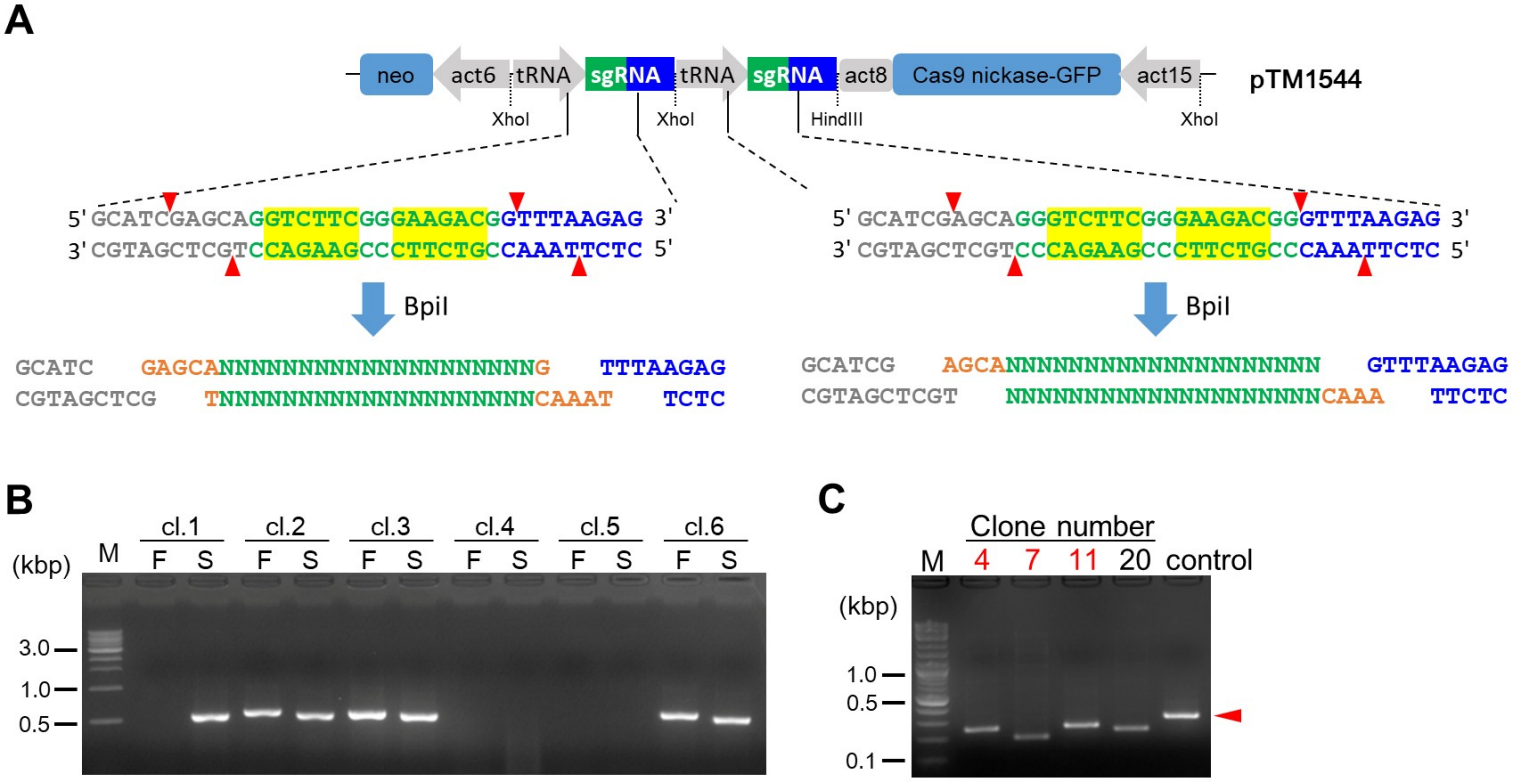
Single-step assembly of a dual sgRNA expression vector and efficient genomic deletion. (A) Schematic of the dual sgRNA expression vector designed for single-step assembly. The yellow highlights indicate the BpiI sites, and the green and orange letters show the target sequences of the sgRNAs and the overhangs for the Golden Gate cloning, respectively. The BpiI sites in both the first and second sgRNA sites generate different overhangs after digestion. act15, act15 promoter; act8, act8 terminator; tRNA, isoleucine tRNA; act6, act6 promoter. (B) Correct insertion of the B1-2 and T1 sgRNA sequences into pTM1544 via one-step cloning. Correct insertion was confirmed via colony PCR. The B1-2 sequence was inserted as the first target (F), and T1 was inserted as the second target (S). (C) Genomic deletions generated via transient introduction of pTM1544. PCR products produced using primers flanking the target sites are shown. The arrowhead indicates product size found in the control (the AX2 genome).

Although an all-in-one vector that enables expression of two sgRNAs and Cas9 nickase was constructed, the generation of these constructs requires two rounds of cloning to insert two sgRNA sequences into dual tRNA-sgRNA cassettes. To achieve simultaneous insertion of two annealed oligonucleotides via one-step cloning, a new dual sgRNA expression vector, pTM1544, was constructed based on a previous report [28]. Both tRNA-sgRNA cassettes contain two BpiI sites; however, unlike the original BpiI sites that generate TGCT and GTTT overhangs, the new sites generate TTTA and GCTC overhangs (Fig 3A). We tested whether we could simultaneously insert two annealed oligonucleotides with different complementary overhangs via one-step cloning. The B1-2 and T1 targets shown in Table 1 were simultaneously inserted into both cloning sites, and half of the analysed clones contained both sgRNA sequences (Fig 3B). These data demonstrated that this new one-step cloning vector allows straightforward generation of Cas9 nickase vectors for dual sgRNA expression.

We next tested whether the dual sgRNA/Cas9 nickase-expressing vector could efficiently induce deletions within the target region. After transient introduction of the vector, 4 out of 21 clones (19%) showed deletions of approximately 100 bp in the first attempt (Fig 3C). Sanger sequencing also confirmed deletions in the target region. These data indicated that the all-in-one Cas9 nickase/dual sgRNA-expressing vector engineered for single-step assembly could facilitate efficient deletion at the desired target site.

### Double nicking-induced HDR for precise genome editing

**Fig 4:**
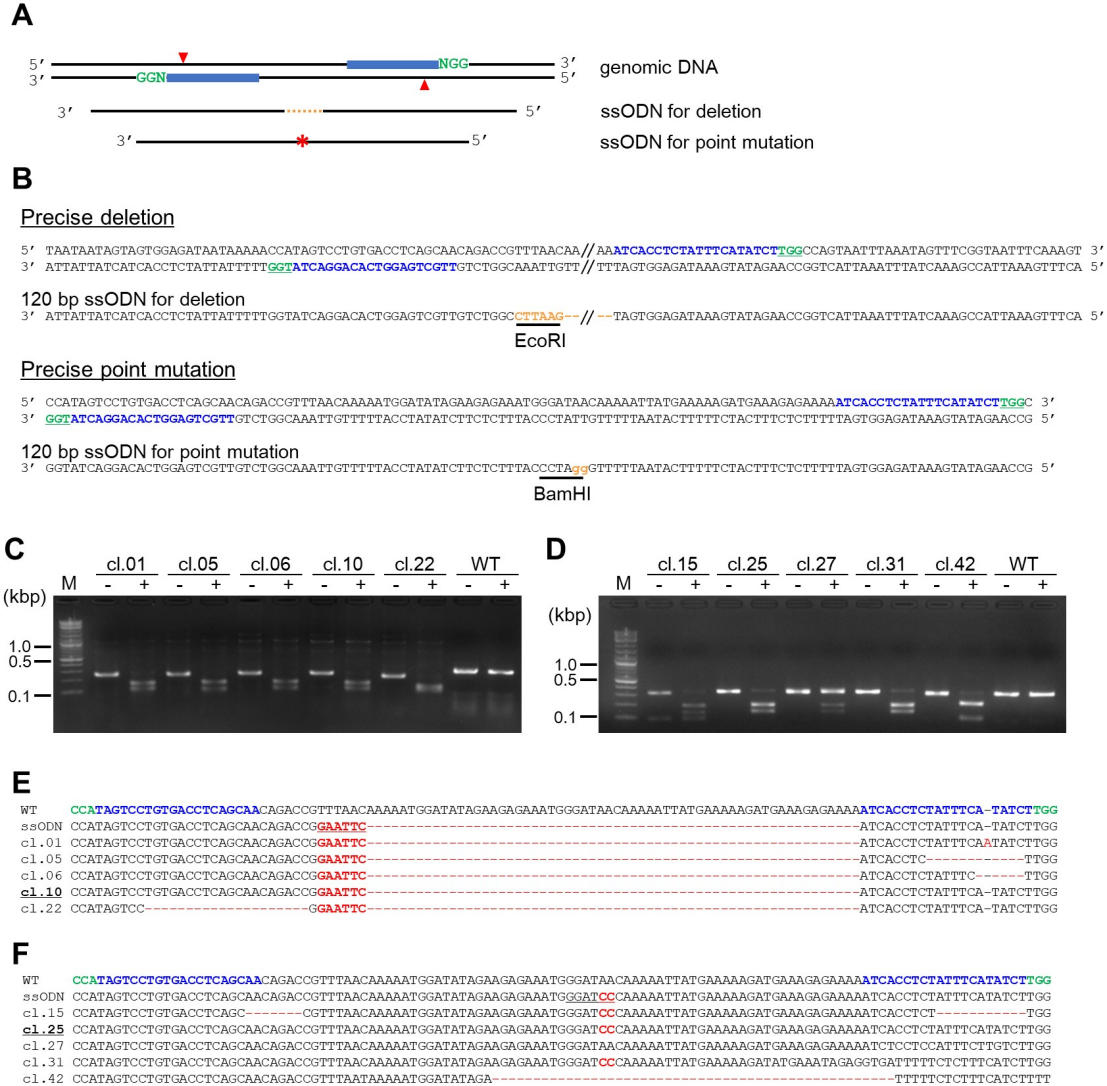
Double nicking-mediated precise genome editing via HDR. (A) Schematic illustration of the precise genome editing approach using paired Cas9 nickases and single-stranded oligonucleotides (ssODNs). Point mutations and deletions are marked by orange broken lines and asterisk, respectively. (B) The sequences used as the ssODN templates. A 66 nt deletion and EcoRI restriction site are shown in orange. Point mutations are indicated by orange lowercase letters and the BamHI site is underlined. (C, D) PCR and restriction enzyme analysis of individual clones. PCR products before (-) and after digestion with the appropriate restriction enzyme (+) are shown. (E, F) The wild-type and mutated *pkaC* sequences. Target sequences are blue and mutations are in red.

It is known that double nicking-induced HDR enables highly precise genome editing [15]. To evaluate double nicking-induced HDR in *D*. *discoideum*, we targeted the *pkaC* gene with pairs of T1 and B1 sgRNAs and introduced ssODN HDR repair templates containing deletions or point-mutations (Fig 4A). One ssODN contained a 66 bp deletion and an EcoRI site, and another contained a point mutation and a BamHI site, and both ssODNs contained appropriate homology arms (Fig 4B). Five pmol of ssODN and a plasmid expressing Cas9 nickase and dual sgRNAs were introduced into the cells, and independent clones were then isolated. The introduction of both a sgRNA pair and a ssODN successfully induced HDR, and 8–23% of the clones showed insertions of EcoRI or BamHI cleavage sites (Fig 4C & 4D). To further characterise the precision of the genome editing, we next analysed the sequences of the target region. A precise deletion and a precise point mutation were observed in clones #10 and #25, respectively (Fig 4E & 4F). In other clones, the expected deletions and point mutations occurred at the target region, although unexpected deletions were also observed near the target sequences. It is notable that clone 27 was a mixture rather than a single clone since the original sequence and a point mutation were both detected via the restriction enzyme assay and sequencing analysis. These data indicated that we successfully generated precise deletions and point mutations in the *D*. *discoideum* genome without the need for integration of a drug resistance cassette by using ssODN and transient introduction of a vector that expresses the Cas9 nickase and two sgRNAs.

## Discussion

Genome editing via CRISPR/Cas9 is widely used as a gene knockout tool in various organisms, while the possibility of creating off-target mutations is a significant concern. In this study, we successfully introduced a deletion of approximately 1.8 kb into the *D*. *discoideum* genome via a double nicking method using Cas9 nickase (D10A), which can reduce the occurrence of off-target effects by 50- to 1,500-fold compared with their prevalence with conventional Cas9-mediated genome editing. The deletion efficiency of our system was higher than that of the conventional Cas9 method. Although such a phenomenon is also observed in mammalian cells [29], Cas9 nuclease, rather than Cas9 nickase, has been widely used in genome editing research. Therefore, it was an unexpected result that the nickase was more efficient in generating deletions. These observations may be due to differences in the kinetics of the DNA repair mechanisms after the binding of Cas9 protein to the target region. In mammalian cells, Cas9-generated mutations are repaired within 1.4–10.7 h [30]. Furthermore, immunofluorescent labelling of transient DSB-dependent foci, i.e., H2A.X foci, revealed that clearance of such foci requires a few hours [31–33]. Such a repair rate has not been precisely measured in *D*. *discoideum*, while the cell cycle checkpoint was released 2–3 h after the induction of DSBs with a low concentration of bleomycin [34, 35], suggesting that the repair rate is similar to that in mammalian cells. Since the NHEJ pathway appears to be involved when dual nicking results in 5' overhangs [18, 19, 36], we assumed that genomic deletions would likely be formed during the time required for the repair of nickase-mediated damage. Of course, the possibility that the nickase binds to the target region more efficiently and induces mutations cannot be excluded.

We have previously reported that simultaneous expression of multiple sgRNAs could successfully introduce indel mutations in multiple genes [6]. Since indel mutations result from the insertion or deletion of several nucleotides, mutants carrying truncated gene products are sometimes created. On the other hand, it is possible to disrupt gene function entirely via the long deletions generated by nickases; thus the nickase approach could be used as an alternative to conventional HR-based gene disruption. Indel and long deletion mutations generated via CRISPR/Cas9 can be used for a wide range of functional analyses, depending on the purpose of the research. In the analysis of essential genes, knockout mutations result in lethality when the gene function is completely disrupted via conventional HR or nickase-mediated deletion. However, it may be possible to isolate indel mutants that express truncated gene products with partially impaired functions. In fact, when we attempted to introduce indel and long deletion mutations into the *ldhA* gene, which encodes an ortholog of D-lactate dehydrogenase, we recovered only indel mutations with 36% (9/25) efficiency.

The relatively short deletions induced at the B1 and T1 targets in *pkaC* resulted in truncated inhibited gene function in some of the mutants, resulting in the cells exhibiting an aggregation-positive phenotype. These targets were designed at the end of the gene to allow analysis of the lengths of the deletions. However, to generate knockout mutations in a particular gene of interest, a long deletion within a functional domain can be created, as is often the case when designing gene knockouts generated via traditional HR. In previous reports, the efficiency of generating deletions longer than 100 bp was low [15, 21]. By contrast, deletion mutations exceeding 1 kb were efficiently generated via our system in *D*. *discoideum*. One potential explanation for this difference is effects of the local chromatin environment, as recruitment of repair factors depends on the chromatin state [37]. Another possibility is differences in the cell cycle, as it is closely related to DNA repair [38]. The cell cycle distribution in *D*. *discoideum* differs from that of mammalian cells, as the majority of *D*. *discoideum* cells are in G_2_ phase [34, 39]. While long deletions were generated relatively easily, deletions sometimes occurred slightly away from the target sequence or nearly complete DNA repair was detected on one side of the targets. These observations imply that other repair pathways, such as microhomology-mediated end joining (MMEJ), or alternative NHEJ (A-NHEJ) can perform the repair [40, 41].

The CRISPR-mediated deletion efficiency varied with the method of sgRNA expression. When two sgRNAs were expressed from two independent pTM1355 vectors, the efficiency was lower than when two sgRNAs were simultaneously expressed from a single pTM1331 vector. Similar results have been observed in some of our other experiments. For example, it was found that the deletion efficiency decreased when the B1 and T1 sgRNAs were cloned individually into the pTM1331 vector (which was originally used as a dual sgRNA expression vector) and expressed in the cells from two separate vectors. In another example, simultaneous expression of five sgRNAs targeting PI3K genes from a single vector was more efficient than a mixture of five individual expression vectors [6]. These data imply that the introduction of several different vectors results in different copy numbers of the sgRNAs in different cells. Moreover, even if the copy number of the vectors is constant, it is likely that there is heterogeneity in the expression levels between cells. It is widely known that there is heterogeneity in gene expression between cells even when they are growing under the same culture conditions [42–44]. Studies of expression vectors revealed that the position and alignment of the expression cassette might also affect the expression efficiency [24, 45, 46]. We noticed that the deletion efficiency was somewhat lower when one of the tRNA-sgRNA cassettes was inserted into the promoter side of the Cas9 nickase gene. Thus, our final vector was designed to align and orient the sgRNA expression cassette to promote high expression to ensure efficient genome editing.

It was shown that introduction of ssODNs along with the Cas9 nickase system generated accurate deletions and nucleotide substitutions in *D*. *discoideum*. A new base editing method has also been developed that introduces point mutations without DSBs via a dCas9-deaminase fusion protein [47–49]. However, this method is limited to base editing to generate C to T or A to G substitutions, and it is challenging to use for precise base editing that requires a high degree of freedom, such as substitution with a different base. Our nickase-based system can more flexibly be used to generate precise point mutations. In *D*. *discoideum*, examples of nucleotide substitutions generated via HR are known, such as a K4A substitution in the histone H3a genomic region [42, 50]. Because the histone H3a gene is relatively short and a blasticidin resistance cassette was successfully introduced downstream of the gene, a substitution mutation could be produced via HR by placing a nucleotide substitution in the homology arm. By contrast, it is not necessary to introduce a drug resistance cassette for CRISPR-mediated generation of precise point mutations; therefore, this approach is useful for introducing point mutations into long genes, such as myosin, which has been extensively studied in *D*. *discoideum* [51, 52].

Similar to our previous Cas9 system, this system does not require the insertion of a drug resistance cassette into the genome; therefore, drug resistance cassettes can be used for other experiments. Other advantages of this system are that the sgRNAs disappear from off-target regions and repeated DNA cleavage does not occur because the vector is only transiently carried by the cells. It is worth mentioning that the mutations generated by this method can be more accurately identified via mutation detection PCR compared with the mutations generated via the previous approach. In addition to these advantages, we have also developed a new dual sgRNA expression vector in which two sgRNA sequences can be simultaneously integrated via one-step cloning, and we believe that this vector will be a powerful tool for genome editing technology in future in *D*. *discoideum*.

## Supporting information

S1 Fig Schematic representation of the new CRISPR/Cas9 vectors. (A) Cas9 and dual sgRNA expression vector, pTM1372. (B) Cas9 nickase and dual sgRNA expression vector, pTM1331. (C) Extrachromosomal vector for Cas9 nickase and sgRNA expression, pTM1355. (D) Cas9 nickase and dual sgRNA expression vector for one-step cloning, pTM1544.
(TIF)

S1 File Original images for gels. (PDF)
