# Supplementary figures and images for: Generation of deletions and precise point mutations in Dictyostelium discoideum using the CRISPR nickase
Source: PLoS One. 2019 Oct 17;14(10):e0224128. doi: 10.1371/journal.pone.0224128 (PMC6797129; doi:10.1371/journal.pone.0224128)

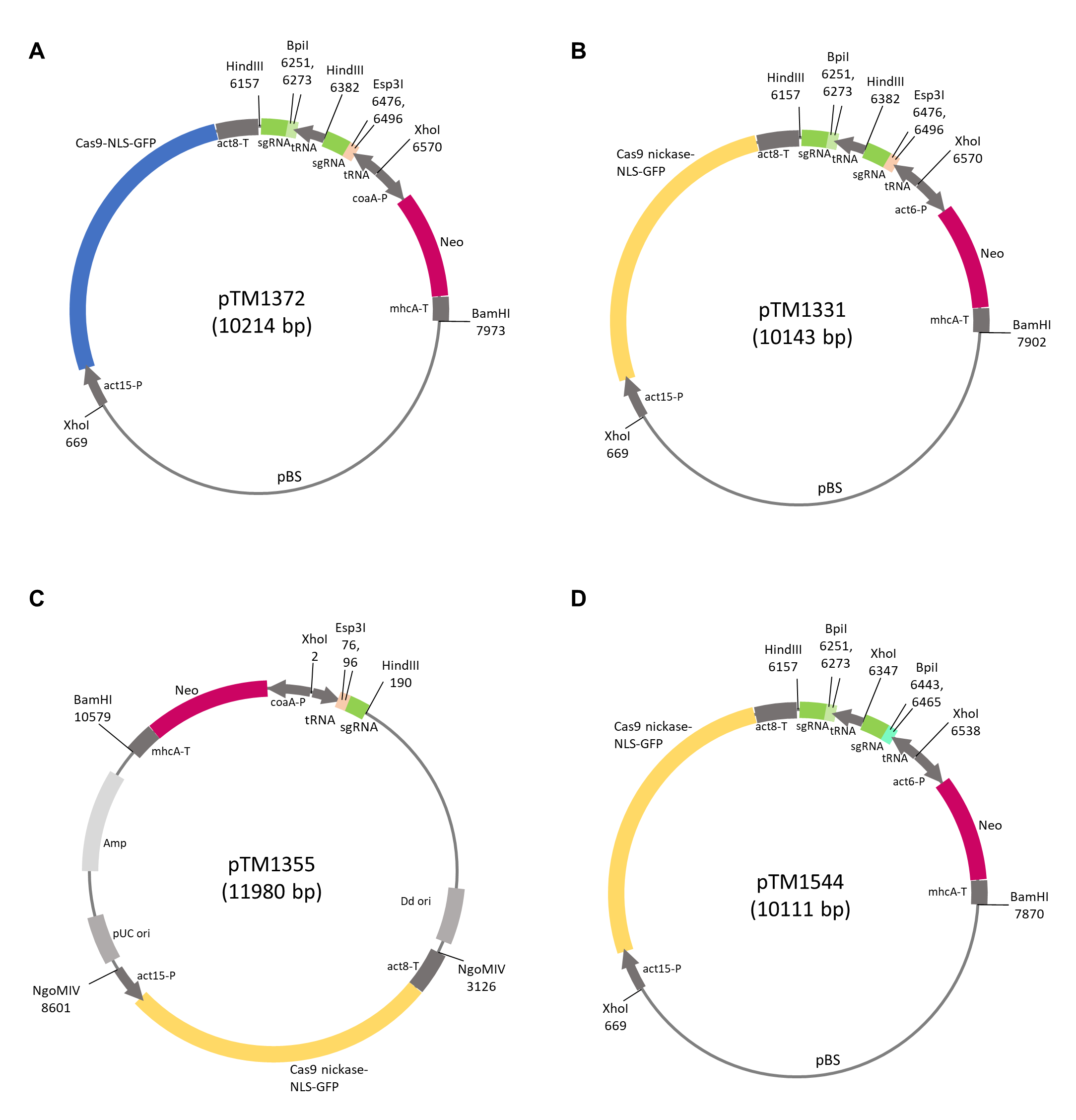

Supplement: S1 Fig — (A) Cas9 and dual sgRNA expression vector, pTM1372. (B) Cas9 nickase and dual sgRNA expression vector, pTM1331. (C) Extrachromosomal vector for Cas9 nickase and sgRNA expression, pTM1355. (D) Cas9 nickase and dual sgRNA expression vector for one-step cloning, pTM1544. (TIF) [file pone.0224128.s001.TIF]
