# Supplementary material for: Generation of deletions and precise point mutations in Dictyostelium discoideum using the CRISPR nickase
Source: PLoS One. 2019 Oct 17;14(10):e0224128. doi: 10.1371/journal.pone.0224128 (PMC6797129; doi:10.1371/journal.pone.0224128)
Supplement: S1 File — (PDF) [file pone.0224128.s002.pdf]

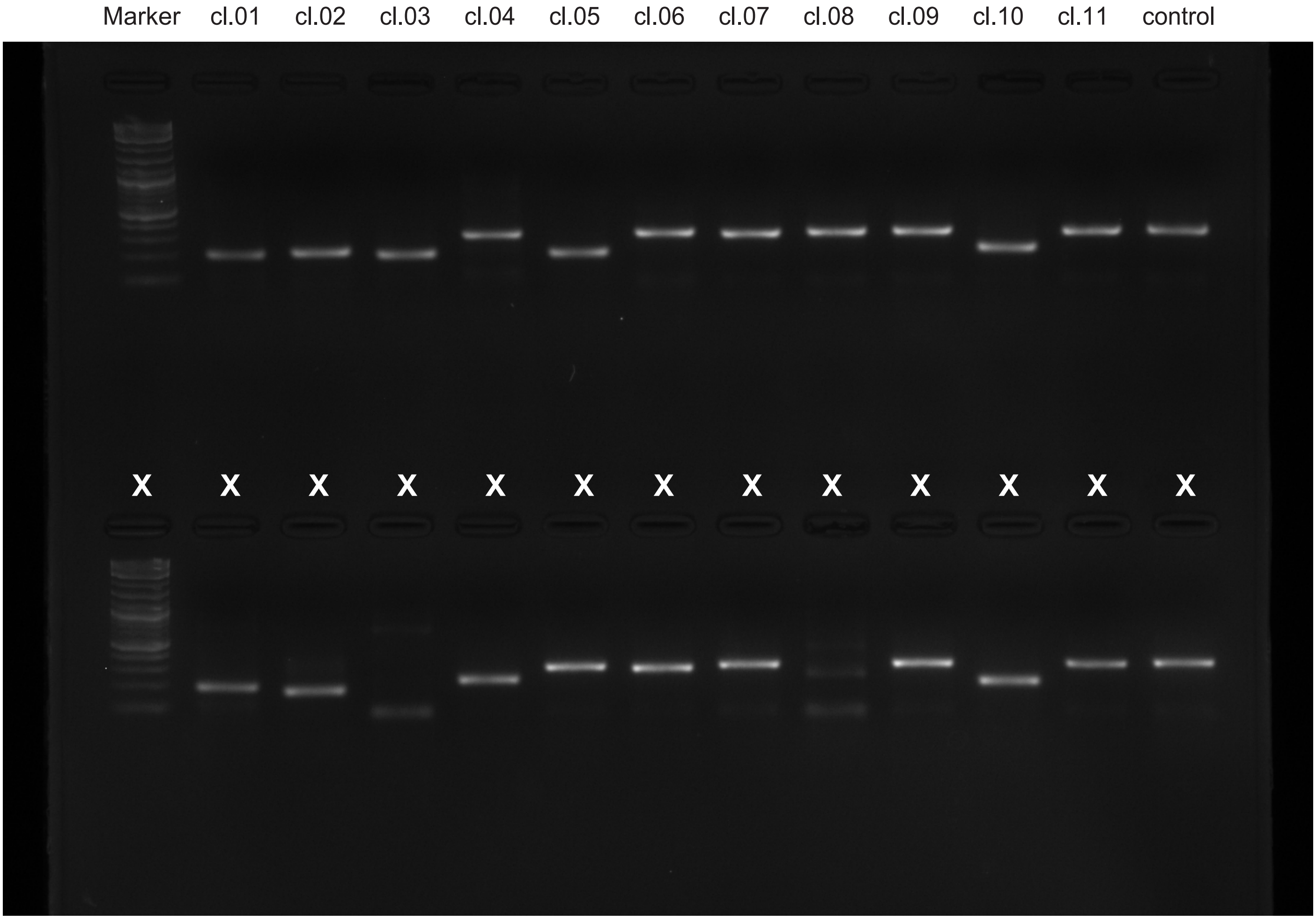

DNA gel was imaged using FAS-V imaging system.

Cropped image is shown in Fig 1C.

Marker 5.3 5.10 5.16 5.21 7.2 7.12 7.16 7.20 8.5 8.11 8.19 control

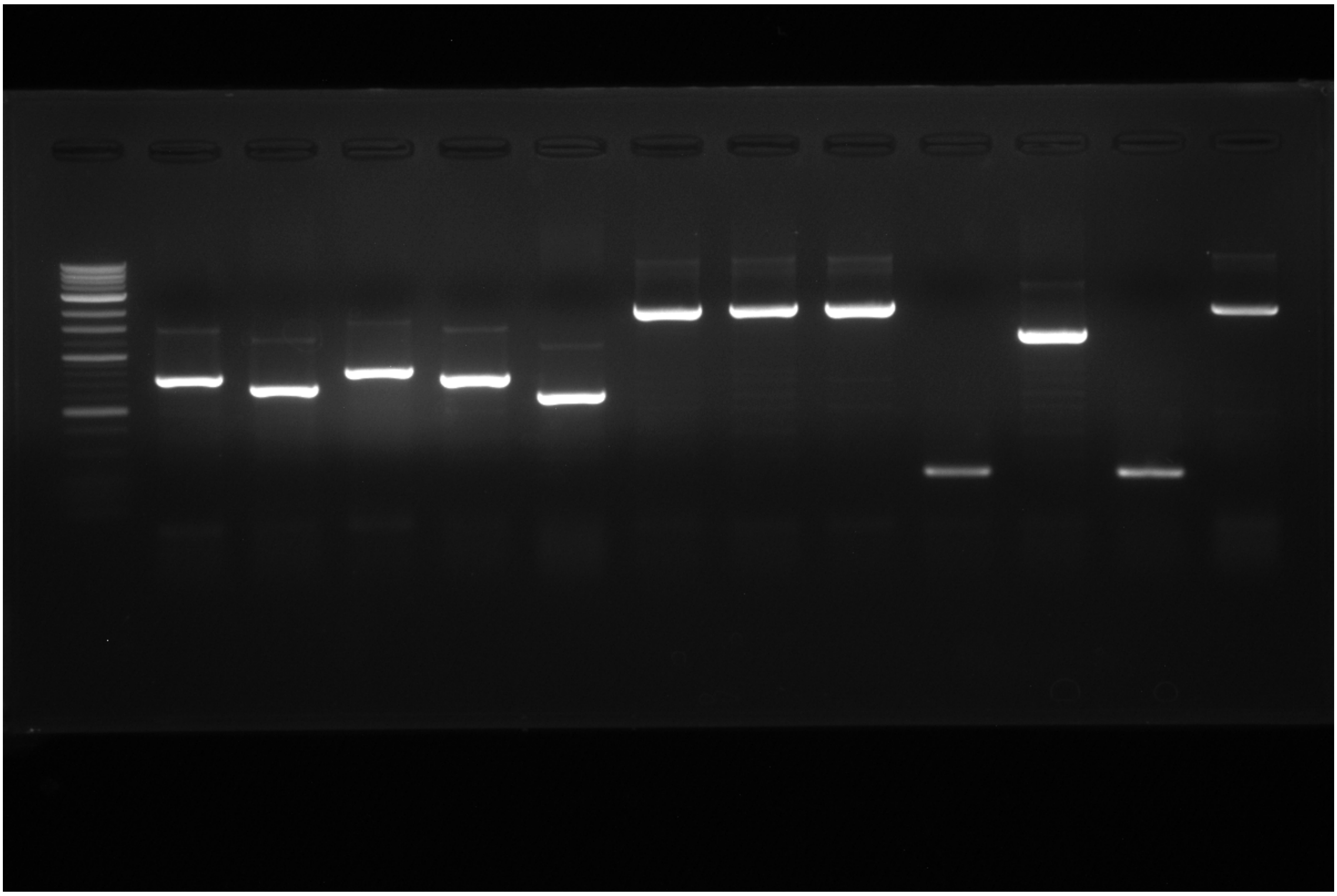

DNA gel was imaged using FAS-V imaging system.

Cropped image is shown in Fig 2C.

Marker cl.1 F cl.1 R cl.2 F cl.2 R cl.3 F cl.3 R cl.4 F cl.4 R cl.5 F cl.5 R cl.6 F cl.6 R

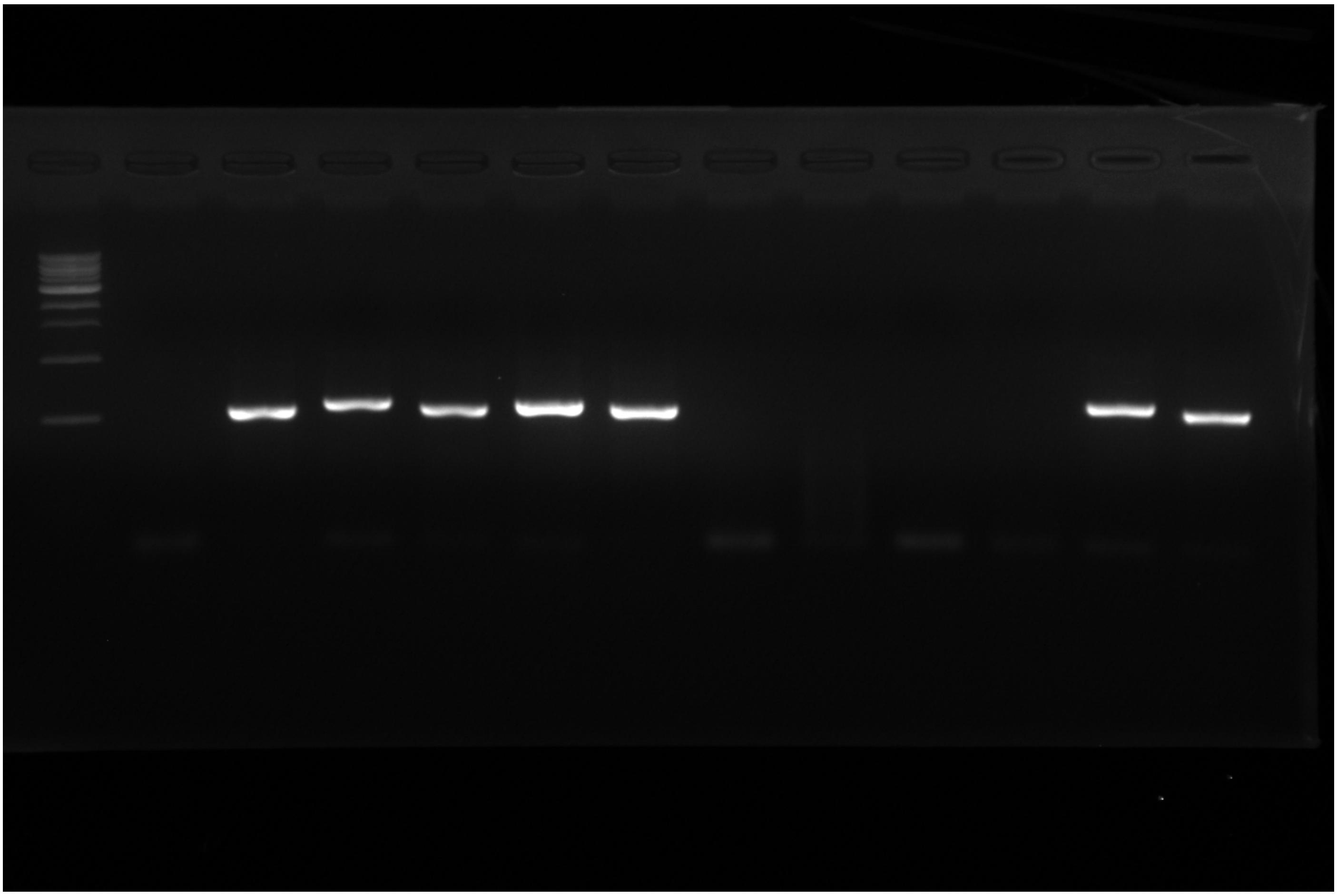

DNA gel was imaged using FAS-V imaging system.

Cropped image is shown in Fig 3B.

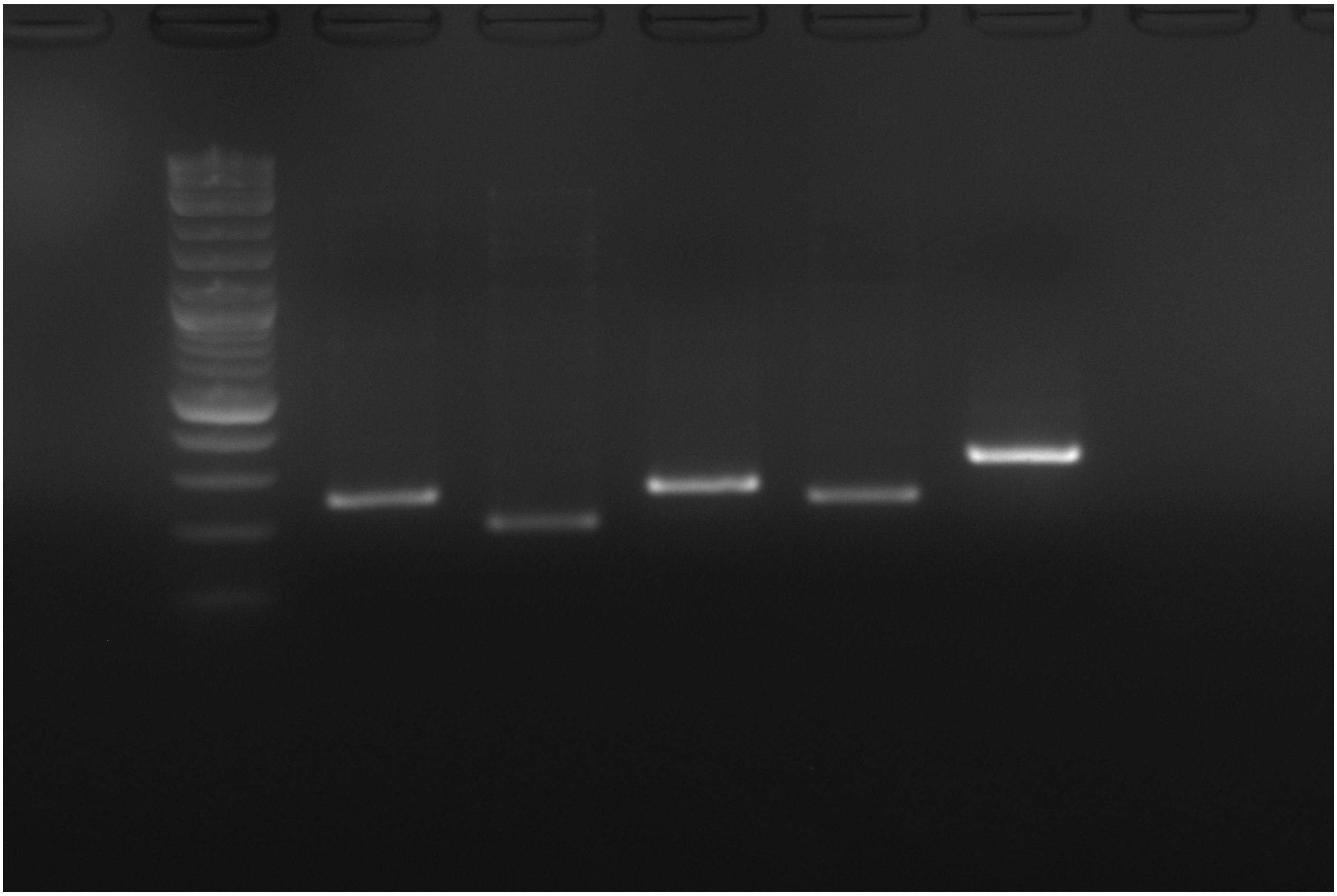

DNA gel was imaged using FAS-V imaging system.

Cropped image is shown in Fig 3C.

Marker cl.01- cl.01+ cl.05- cl.05+ cl.06- cl.06+ cl.10- cl.10+ cl.22- cl.22+ WT- WT+

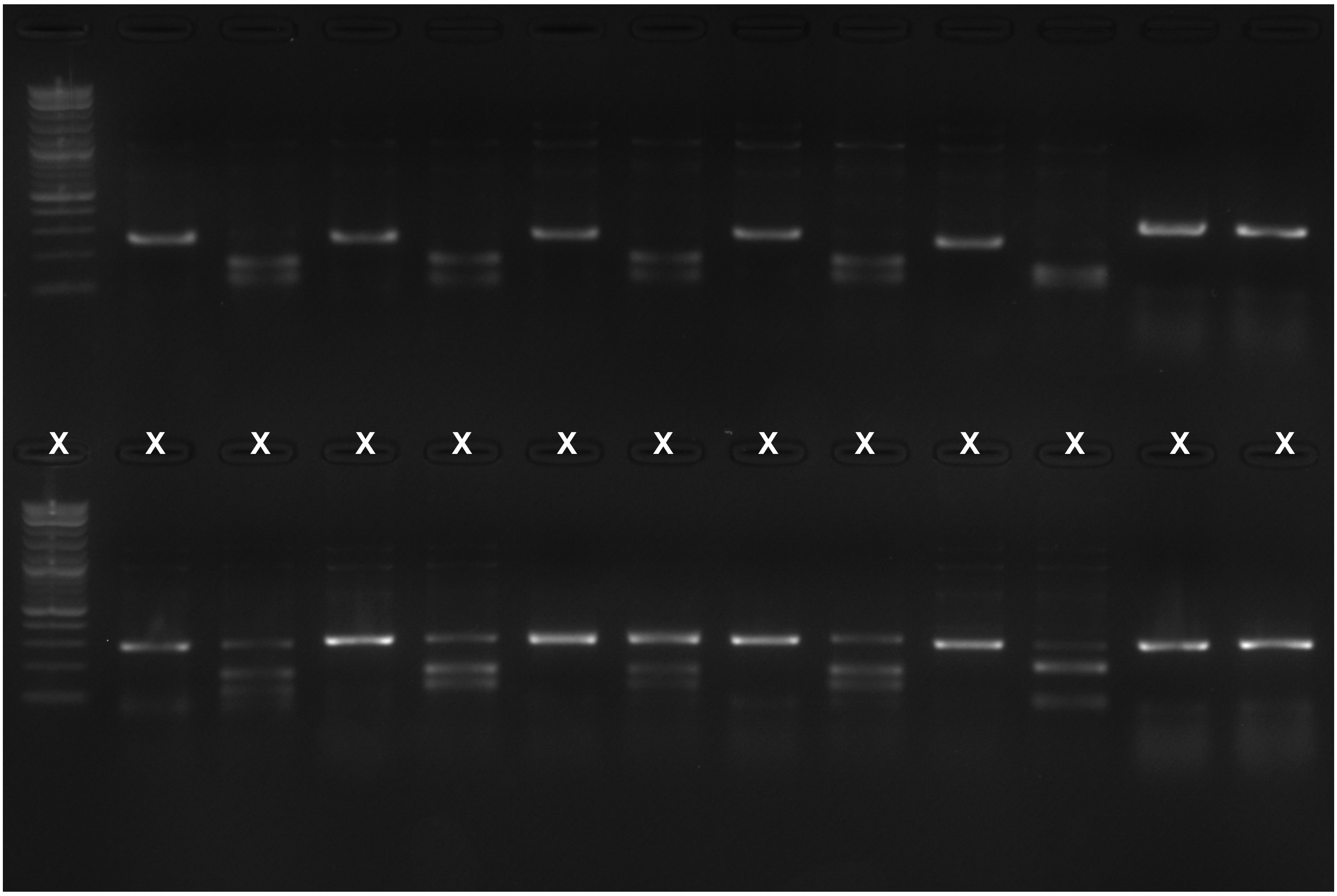

DNA gel was imaged using FAS-V imaging system.

Cropped image is shown in Fig 4C.

Marker cl.15- cl.15+ cl.25- cl.25+ cl.27- cl.27+ cl.31- cl.31+ cl.42- cl.42+ WT- WT+

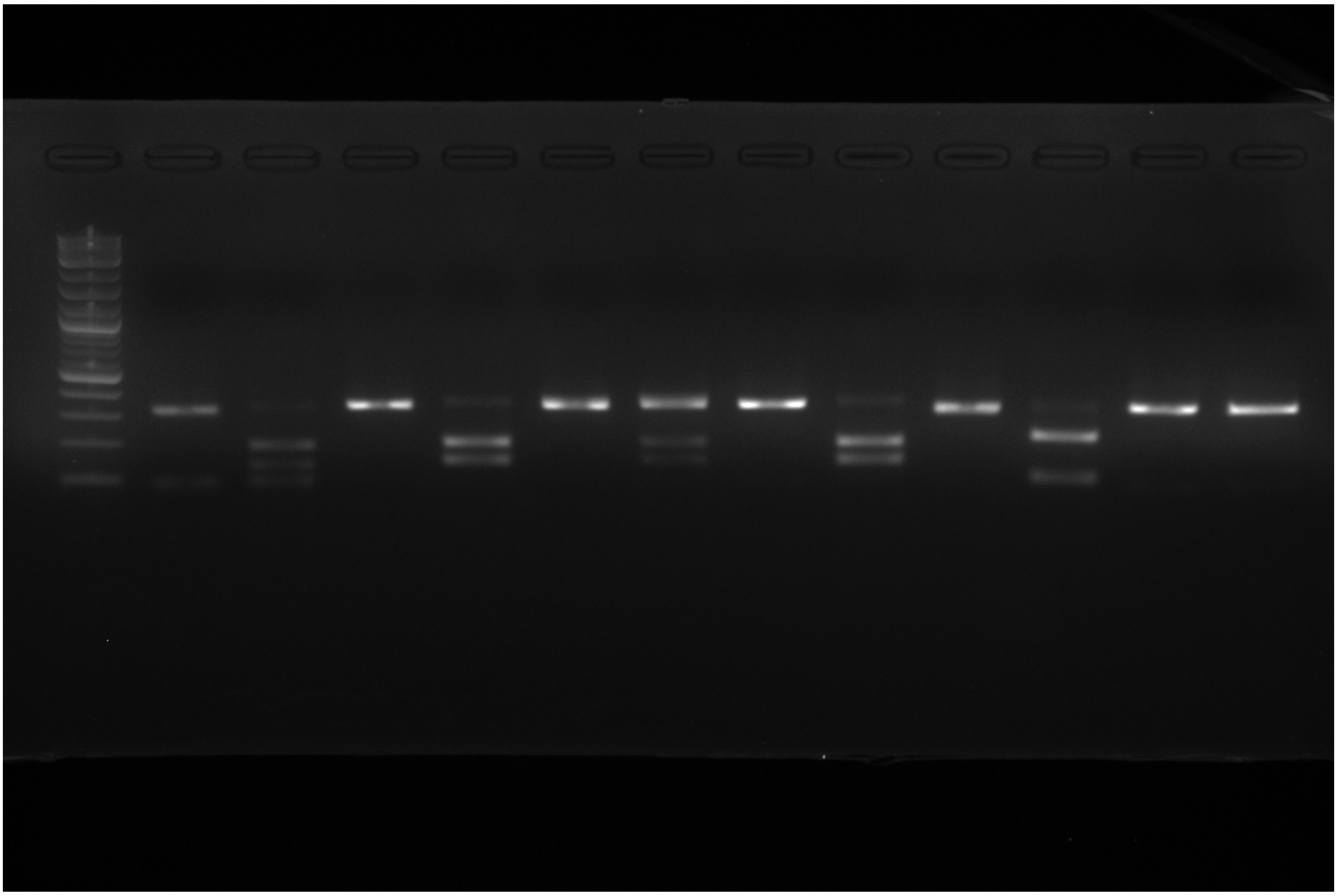

DNA gel was imaged using FAS-V imaging system.

Cropped image is shown in Fig 4D.
